# Comparison of 30 Cytokines in Human Breast Milk between 1989 and 2013 in Japan

**DOI:** 10.3390/nu15071735

**Published:** 2023-04-01

**Authors:** Tomoki Takahashi, Hiroshi M. Ueno, Fumiya Yamaide, Taiji Nakano, Yuki Shiko, Yohei Kawasaki, Chisako Mitsuishi, Naoki Shimojo

**Affiliations:** 1 Research and Development Department, Bean Stalk Snow Co., Ltd., Saitama 350-1165, Japan; 2 Department of Pediatrics, Graduate School of Medicine, Chiba University, Chiba 260-8670, Japan; 3 Biostatistics Section, Clinical Research Center, Chiba University Hospital, Chiba 260-8677, Japan; 4 Faculty of Nursing, Japanese Red Cross College of Nursing, Tokyo 150-0012, Japan; 5 Japanese Red Cross Tokyo Katsushika Perinatal Center, Tokyo 125-0051, Japan; 6 Center for Preventive Medical Sciences, Chiba University, Chiba 263-8522, Japan

**Keywords:** colostrum, human milk, osteopontin, temporal expression of cytokines

## Abstract

Milk cytokines play a vital role in mucosal immunity during infancy by supporting immune development and functions. Although the maternal background characteristics influence milk cytokines, changes in cytokine levels across generations remain unclear. Colostrum (C, *n* = 48) and mature milk (MM, *n* = 49) samples were collected from lactating Japanese women in 1989 (2727 samples) and 2013 (1408 samples). Milk cytokines were comprehensively measured using a suspension array and immunosorbent assays. The positive rates and cytokine concentrations were compared between the two generations using logistic and multiple regression analyses. Twenty-eight cytokines tested positive in all sample groups (1989-C, 1989-MM, 2013-C, and 2013-MM). The median osteopontin (OPN) level was significantly higher in the 1989-C group than in the 2013-C group (318.1 vs. 137.5 μg/mL; *p* = 0.0016) but did not differ between the MM groups. The median TGF-β1 level was significantly lower in the 1989-MM group than in the 2013-MM group (1056.2 vs. 1330.8 pg/mL; *p* = 0.008) but did not differ between the C groups. Most cytokines were comparable between generations, except for potential variation in the C-OPN and TGF-β1 levels. Milk cytokine secretion may reflect temporal changes in maternal background characteristics; however, the results from the analysis of 30-year-old samples may have influenced the milk cytokine levels. Further studies are needed with a larger number of milk samples collected from the same individuals at multiple time points over a wide lactation period, with detailed data on the maternal and infant background characteristics and diets.

## 1. Introduction

Breast milk contains the fundamental nutrients required by infants, including proteins, fats, minerals, and vitamins, as well as a large variety of bioactive compounds. Cytokines are one of the major classes of compounds that play an important role in mucosal immunity during infancy by operating in networks and orchestrating immune system development and functions [1].

Several reports have confirmed that the cytokines in breast milk can influence infant health outcomes [2,3,4,5]. Osteopontin (OPN) is a cytokine that accounts for approximately 2.1% of the total protein in human breast milk [6] and affects immune functions and intestinal development in infants. OPN has been shown to play a role in a variety of physiological processes, including inflammation, immune responses, and tissue repair. OPN is relatively resistant to gastrointestinal digestion and can be found in other body fluids, including blood and urine [6,7,8]. Compared with infants who were fed regular formula, infants that were fed formula with added bovine milk OPN showed significantly reduced serum levels of tumor necrosis factor-α (TNF-α) and fewer days of fever [2]. Furthermore, the immune cell profile of infants who received OPN was reported to be similar to that of breast-fed infants [9]. Higher levels of transforming growth factor-β (TGF-β) in breast milk are associated with decreased rates of atopic dermatitis and asthma in infants [3]. In addition, increased levels of interleukin (IL)-1β in breast milk are associated with a reduced risk of eczema in early childhood [4]. Eotaxin may be a biomarker for the development of atopic dermatitis in infancy [5]. However, high inter- and intra-individual variations lead to inconsistent findings among studies. Recent research suggests that the milk cytokine profile reflects genetic and environmental factors [10,11,12,13,14]. Maternal background characteristics, such as diet, physical characteristics, and socioeconomic status, have changed over time; thus, the breast milk composition may have changed as well.

Recent studies have shown that the vitamin D and 25-hydroxyvitamin D (25OHD) concentrations in Japanese breast milk were lower in 2016–2017 than in 1989 [15]; this might have been because of a decrease in maternal exogenous and endogenous vitamin D due to decreased maternal fish intake and UV exposure [16]. Vitamin D receptors are expressed on many immunologically active cells, especially regulatory T cells; the individual cytokines produced by these cells operate in networks to produce a cascade of effects that contribute to the orchestration, development, and functions of the immune system [17]. Therefore, maternal vitamin D depletion over time may affect the cytokine profile of breast milk by altering the maternal immune response. However, there are no reports on decennial changes in the cytokine profile of breast milk.

In this study, we hypothesized that the cytokine profile of human breast milk would be affected by changes in the maternal background characteristics that occurred across generations. We measured the changes in 30 cytokines in breast milk samples collected from mothers of different generations.

## 2. Materials and Methods

### 2.1. Study Design

This comparative study used samples from studies conducted in 1989 and 2013. In 1989, a nationwide cross-sectional study was conducted on the composition of breast milk in healthy lactating Japanese women; milk samples were collected across 29 prefectures [18]. In 2013, a 2 × 2 factorial, randomized, non-treatment, controlled trial was conducted in the Tokyo metropolitan area to evaluate the prevention of atopic dermatitis or food allergies via a skin care regimen and synbiotics in infants (not in mothers) [19]. This study was conducted in accordance with the recommendations of the Ethical Guidelines for Clinical Research (Ministry of Health, Labour, and Welfare, Japan) and was approved by the Ethics Committee of the Faculty of Medicine, University of Chiba (Chiba, Japan; reference No. 2067). All participants provided written informed consent in accordance with the Declaration of Helsinki. This study was registered with the University Hospital Medical Information Network Clinical Trials Registry (JPRN-UMIN000010838), https://center6.umin.ac.jp/cgi-open-bin/ctr/ctr_view.cgi?recptno=R000012665.

### 2.2. Collection of Human Milk Samples

In this study, 194 breast milk samples were randomly included from the milk samples collected during two separate studies conducted in 1989 and 2013. These comprised colostrum (C; produced within 7 days of birth; 1989: *n* = 48, 2013: *n* = 49) and mature milk (MM; produced 20–38 days postpartum; 1989: *n* = 48, 2013: *n* = 49) samples. The discrepancy in the numbers of C and MM samples was attributed to the limited number of assay plate wells, which will be described later. During the 1989 study, 2727 breast milk samples were collected from 2434 lactating women (17–41 years old) nationwide in Japan [18]. Mothers collected the milk samples in plastic tubes using a breast pump after breastfeeding. The participant characteristics in the 1989 study were “maternal age (years)”, “maternal body mass index (BMI; kg/m^2^)”, “infant age (days)”, and “infant birth weight (g)”. Conversely, during the 2013 study, 1408 samples (486 C samples, 490 MM samples, and 432 MM samples at 3 months postpartum) were collected from 605 lactating women (24–45 years old) living in metropolitan areas around Tokyo and Chiba [19]. The participant characteristics in the 2013 study were “maternal age (years)”, “maternal BMI (kg/m^2^)”, “maternal primiparity”, “maternal allergy status”, “maternal smoking status”, “infant age (days)”, “gestational age (weeks)”, “infant birth weight (g)”, “cesarean section (C-section)”, “infant sex”, and “infant allergy status”. The 1989 and 2013 study samples were properly stored at −80 °C within 12 h of collection and were never thawed throughout the storage period due to unexpected temperature problems (e.g., breakdown or mechanical failure of the refrigerators). The total storage periods from sample collection to the measurement date were approximately 5 years for the 2013 study and 28 years for the 1989 study. The C and MM samples were only thawed and centrifuged using the appropriate method prior to each assay performed in this study [6,20,21].

### 2.3. Multiplex Analysis

A Bio-Plex Pro™ Human Cytokine 27-plex Assay (Bio-Rad Laboratories, Inc., Tokyo, Japan) was used to measure the cytokines in the human breast milk samples. The samples were analyzed for IL-1β; IL-1 receptor antagonist (RA); IL-2; IL-4; IL-5; IL-6; IL-7; IL-8; IL-9; IL-10; IL-12 (p70); IL-13; IL-15; IL-17; eotaxin; fibroblast growth factor-basic (FGF-basic); granulocyte colony-stimulating factor (G-CSF); granulocyte macrophage colony-stimulating factor (GM-CSF); interferon-gamma (IFN-γ); interferon-gamma induced protein-10 (IP-10); monocyte chemotactic protein-1 (MCP-1); macrophage inflammatory protein-1α (MIP-1α); MIP-1β; platelet-derived growth factor-BB (PDGF-BB); regulated on activation normal T cell expressed and secreted (RANTES); TNF-α; and vascular endothelial growth factor (VEGF). Before measuring the cytokine levels, a minimum of 100 μL of each milk sample was immediately thawed and centrifuged, first at 500× *g* for 5 min and then at 10,000× *g* for 10 min, using a Thermo Sorvall Legend XTR Refrigerated Centrifuge (Thermo Fisher Scientific, Inc., Tokyo, Japan). The resulting skimmed milk was collected using pipettes, and assays were performed according to the manufacturer’s instructions [22]. A total of 194 samples (1989-C, *n* = 48; 1989-MM, *n* = 48; 2013-C, *n* = 49; 2013-MM, *n* = 49) were randomly distributed into five plates and assayed using the same set of kits. Each 96-well plate included 39 human milk samples, 8 standards, and 1 blank well in duplicate. The cytokine levels were converted to concentrations using a five-parameter logistic model that was generated using each cytokine from the sample plates. The limit of detection (LOD) was calculated separately for each plate. Cytokines below the LOD were judged to be negative, and a specific concentration of the LOD was allocated for each cytokine to aid the statistical analysis. Of the cytokines that were judged to be positive, those below the lower limit of quantitation (LLOQ) were allocated the concentration of the LLOQ to aid the statistical analysis.

### 2.4. Measurement of OPN, TGF-β1, and sCD14

OPN, TGF-β1, and sCD14 were measured using a Human Osteopontin Quantikine ELISA Kit, a Human TGF-beta 1 Quantikine ELISA Kit, and a Human CD14 Quantikine ELISA Kit, respectively (R&D Systems Inc, Minneapolis, MN, USA). Before the analysis, the milk samples were thawed and centrifuged to remove residual cream or precipitate as per the assay manufacturer’s instructions [6,20,21] and as described previously [20,21,23]. The cytokine levels were converted to concentrations using a five-parameter logistic model. The LLOQs for OPN, TGF-β1, and sCD14 were 0.31 ng/mL, 31.3 pg/mL, and 250 pg/mL, respectively. Cytokines below the LLOQ were judged to be negative and were allocated the concentration of the LLOQ to aid the statistical analysis.

### 2.5. Data Analyses

Data are presented as medians for continuous variables (cytokine levels and their lactational change ratios from C to MM) and as numbers with percentages for categorical variables (positive results) due to the non-normal distributions of most of the outcome data. Categorical variables were compared between two groups using Fisher’s exact test. All continuous variables were compared between two groups using a Mann–Whitney U test owing to the non-normal distributions. During the multivariate analysis, a logistic regression analysis was performed for dichotomous outcomes after adjusting for “maternal age (year)” and “infant birth weight (g)” as predisposing variables. When there was a complete or quasi-complete separation in the logistic regression analysis, the Firth correction method was applied. A multiple regression analysis, with rank order as the dependent variable, was also performed to compare continuous outcomes after adjusting for the “maternal age (year)” and “infant birth weight (g)”, as in the logistic regression.

Fisher’s exact test and the Mann–Whitney U test were performed using SPSS Statistics (version 26; IBM Inc., Chicago, IL, USA). The logistic regression analysis and multiple regression analysis (with rank order as the dependent variable) were performed using SAS (version 9.4; SAS Institute, Cary, NC, USA). *p*  <  0.05 was the criterion for significance.

## 3. Results

### 3.1. Participant Characteristics

Maternal age was significantly higher in 2013 than in 1989 for the C and MM groups (Table 1). Infant age was significantly lower in 2013 than in 1989 for the C and MM groups. Infant birth weight was significantly lower in 2013 than in 1989 in the MM group. The other characteristics could not be compared between the 1989 and 2013 samples due to the unavailability of data for the 1989 samples.

### 3.2. C Samples

Twenty-eight cytokines were present in the C samples. The serum levels of IL-1β (*p* = 0.038), IL-17 (*p* = 0.007), IFN-γ (*p* = 0.032), IL-5 (*p* = 0.016), IL-2 (*p* = 0.031), IL-15 (*p* = 0.028), and FGF-basic (*p* = 0.005) were significantly higher in 2013 than in 1989 (Table 2). However, these differences were not significant after the data were adjusted for maternal age and infant birth weight. The IL-1β (*p* = 0.002), IL-17 (*p* = 0.035), IL-8 (*p* = 0.009), MIP-1α (*p* = 0.0002), FGF-basic (*p* = 0.024), and G-CSF (*p* = 0.0003) levels were significantly higher in 2013 than in 1989, but only OPN (*p* = 0.00002) was significantly higher in 1989 than in 2013. After adjusting for maternal age and infant birth weight, these differences were not significant, except for the differences in IL-1β (*p* = 0.038) and OPN (*p* = 0.0016).

### 3.3. MM Samples

Twenty-seven cytokines were present in the MM samples. No significant differences in the positive rates of cytokines were detected between 1989 and 2013 (Table 3). The IL-7 (*p* = 0.017) and IP-10 (*p* = 0.023) levels were significantly higher in 1989 than in 2013 when adjusted for maternal age and infant birth weight, although no significant differences were detected prior to the adjustments. The TGF-β levels (*p* = 0.008) were significantly higher in 2013 than in 1989 when adjusted for maternal age and infant birth weight, although no significant differences were detected prior to the adjustments.

### 3.4. Lactational Change Ratios of Cytokines

Lactational changes were additionally analyzed for the selected cytokines, incorporating data based on the thresholds of two-fold and above or less than half-fold in the concentration ratios (Table 4). As the infant age increased, the IL-1β (*p* = 0.021), TNF-α (*p* = 0.039), IP-10 (*p* = 0.007), and MIP-1α (*p* = 0.012) levels decreased more significantly in 2013 than in 1989 (Table 3). The OPN levels decreased as the infant age increased in 1989 and increased as the infant age increased in 2013.

## 4. Discussion

This was the first study to evaluate multiple immune components in C and MM samples collected from different generations of mothers in a Japanese general population. The OPN concentration in C samples was found to be significantly lower in 2013 than in 1989, and it changed differently as the infant age increased in 1989 and 2013. This study describes the effects of generational changes on the immune profile of human breast milk.

The OPN level detected in C samples was lower in 2013 than in 1989 (Table 2; results of the Mann–Whitney U test and multiple regression, Table S1). This may be explained by differences in sunlight exposure and delivery routes among the different generations that were analyzed. Indeed, the vitamin D content in the milk was higher in the 1989 cohort than in a cohort sampled in 2016–2017, possibly due to limited sunlight exposure in the recent mothers [15]. Furthermore, it was reported that the OPN content was lower in the breast milk samples from mothers who delivered via a C-section than from mothers who delivered vaginally [24]. However, data from the 2013 cohort indicated that the OPN levels were significantly lower in C samples (3–5 days postpartum) from women who delivered vaginally than from women who delivered via a C-section; no differences in the MM samples were noted between the two groups (22–28 days postpartum (Table S2)). Notably, the proportion of pregnant women undergoing C-sections is steadily rising in Japan [25]. A previous study revealed that the OPN levels in breast milk (3 months postpartum) were affected by maternal background characteristics, such as dietary intake (especially energy and fiber intake), BMI, and smoking; in particular, higher OPN levels were found in mothers who delivered vaginally than in those who delivered via a C-section [24]. Oxytocin promotes the expression of the *OPN* gene [26]; its expression is higher during vaginal births than during C-sections [27]. These findings may explain the higher OPN content in milk from women delivering vaginally than from those delivering via C-sections. Accordingly, lifestyle changes would affect the milk OPN content in Japanese lactating women. Furthermore, the concentrations of breast milk components may also differ among samples collected from different generations of mothers due to different extents of protein denaturation with different storage periods. Few studies have examined the effects of different storage periods and temperatures on cytokine concentrations in breast milk; a previous study reported that storage at −80 °C for 12 months reduced the concentrations of IgA, IL-8, and TGF-β1 [28]. Therefore, the cytokine concentrations may be lower in samples collected in 1989 than in samples collected in 2013. Our study revealed that the presence and concentration of cytokines in the breast milk, except for OPN, were lower in 1989 than in 2013; the effects of long-term storage and other chronological factors on breast milk composition are difficult to determine (Table 2; results of Fisher’s exact test, Mann–Whitney U test, and multiple regression). However, the OPN levels were significantly higher in the C samples from 1989 than in those from 2013, even after adjustment for maternal age and infant birth weight. In this study, the OPN concentrations in C samples from 2013 were similar to the previously reported values, indicating that our study results are reliable [23]. From a structural viewpoint, OPN is an intrinsically disordered protein with no specific higher-order structure [29]. Therefore, the OPN in breast milk samples collected in 1989 was likely unaffected by structural changes due to long-term storage. In addition, the OPN levels remained within 162.4–313.9 μg/mL in the pooled milk samples at eight lactational stages (3–482 days postpartum) in the 1989 study [18]. The differences in the OPN levels in the C samples in this study may reflect changes in the maternal immune response from 1989 to 2013. The presence and concentration of other milk cytokines differed between the two generations as well, which may reflect the varied stability of the individual cytokines.

In the samples collected in 2013, the OPN levels increased as the infant age increased (Table 4). The concentration of OPN in the MM samples did not differ significantly between 1989 and 2013 (Table 3; results of the Mann–Whitney U test and multiple regression analysis). Thus, the differences in the OPN concentration patterns may be due to differences in the OPN concentrations in the C samples between the two generations (Table 2). The concentrations of milk proteins, including OPN, generally decrease throughout the lactational period [30,31,32]. Similarly, in Japanese mothers, the OPN level reportedly decreased through lactational changes after 1 month postpartum; however, there are no reports on a lactational change in the OPN level within 1 month postpartum [23]. Because the C and MM samples in our study were collected from different mothers, the lactational change in the OPN levels within 1 month postpartum in 2013 may be a coincidental finding. In a previous study on another cohort comprising a Japanese general population sampled in 2007–2008, we found no significant differences between the C and MM samples collected from the same 49 mothers [33]. Therefore, the lactational changes within 1 month postpartum that were observed in 2013 may be a characteristic of that generation of Japanese mothers. OPN is a key component of various physiological processes in early infancy, such as immune function maturation and intestinal and brain development [2,6,34,35]. In a recent study, the endogenous plasma OPN levels in infants were found to change in sync with the breast milk OPN levels (at 1, 4, and 6 months) [32]. Therefore, the differences in the lactational change patterns observed in this study might have affected the infants’ endogenous plasma OPN levels.

A limitation of this study is that the observed differences might be due to potential confounders, such as maternal (diet, BMI, parity, episodes of allergy, and smoking) and infant (feeding methods, gestational age, delivery route, sex, and episodes of allergy) factors that may affect lactational changes and breast milk components. We could not adjust for such confounders due to insufficient background characteristics in the 1989 study; information on the dietary intakes of both generations was unavailable for this study. Collectively, the delivery route may influence the lactational changes in breast milk OPN levels. For future comparative studies, breast milk samples should be collected from the same individuals at multiple time points over a wide lactation period, in addition to detailed information on the maternal and infant background characteristics and diet; the samples can be stored for several decades to explore the generational variation in unknown elements discovered in the future. Additionally, another study found no associations between breast milk cytokines other than OPN and key maternal background characteristics (including the delivery route), suggesting that the maternal genotype may also be an important determinant or modulator [36].

## 5. Conclusions

We compared the presence and concentrations of 30 cytokines among human milk samples collected from mothers of different generations. Most cytokines remained detectable in breast milk stored at −80 °C for about three decades. Clinically, donor human milk has been reported to be effective in preventing necrotizing enterocolitis, reducing feeding intolerance, and improving the long-term outcomes of premature infants. Some recommendations for the optimal storage conditions in human milk banks and neonatology units have focused on microbiological safety. This study provides new data on the aspects of storage stability of bioactive immunological components. The OPN concentrations in the C samples were significantly lower in 2013 than in 1989, and different lactational patterns of OPN were noted in the samples collected at the two timepoints. These results suggest that generational differences in the maternal background characteristics influence the immune composition of human breast milk, providing a better understanding of the associations between the two.

## Figures and Tables

**Table 1:** Background characteristics of mothers and infants in this study.

	C Samples					MM Samples				
	1989 (*n* = 48)		2013 (*n* = 49)		*p* Value	1989 (*n* = 48)		2013 (*n* = 49)		*p* Value
Mothers										
Maternal age (years), median (IQR)	27	(25.0–29.0)	36	(29.4–40.0)	<0.001 *	28	(25.0–30.0)	37	(31.3–38.3)	<0.001 *
BMI (kg/m^2^), median (IQR)	NA		20.8	(19.3–22.3)	-	NA		20.5	(19.4–21.3)	-
Primiparity, *n*/total (%)	NA		27/49	(55%)	-	NA		20/49	(41%)	-
Allergy, *n*/total (%)	NA		31/49	(63%)	-	NA		34/49	(69%)	-
smoking, *n*/total (%)	NA		1/49	(2%)	-	NA		0/49	(0%)	-
Infants										
Infant age (days), median (IQR)	5	(4–5)	4	(3–5)	0.004 *	31	(30–31)	25	(22–28)	<0.001 *
Gestational age (weeks), median (IQR)	NA		39	(38–40)	-	NA		39	(38–40)	-
Birth weight (g), median (IQR)	3150	(2852–3400)	3044	(2913–3296)	0.395	3159	(2963–3400)	2974	(2777–3224)	0.009 *
C-section, *n*/total (%)	NA		16/49	(33%)	-	NA		16/49	(33%)	-
Sex, male, *n*/total (%)	NA		28/49	(57%)	-	MA		24/49	(49%)	-
Allergy, *n*/total (%)	NA		30/49	(61%)	-	NA		34/49	(69%)	-

Differences in variables between 1989 and 2013 were examined using a Mann–Whitney U test. Dichotomous variables were compared by using Fisher’s exact test. Abbreviations: BMI, body mass index; IQR, interquartile range; NA, not available; C-section, cesarean section; C, colostrum; MM, mature milk. * *p* < 0.05.

**Table 2:** Cytokine profile in the C samples.

	1989 (*n* = 48)		2013 (*n* = 49)		*p* Value			
	Positive Results (%)	Median (IQR)	Positive Results (%)	Median (IQR)	Fisher’s Exact Test	Logistic Regression	Mann–Whitney U Test	Multiple Regression
Anti-inflammatory cytokines								
IL-1ra	100	363.8 (114.0–897.7)	100	573.2 (195.8–1307.9)	-	-	0.086	0.845
Proinflammatory cytokines								
IL-1β	64.6	0.3 (0.1–1.0)	83.7	1.3 (0.3–5.2)	0.038 *	0.117	0.002 *	0.038 *
IL-6	77.1	7.6 (1.4–21.6)	87.8	9.5 (4.4–19.0)	0.191	0.891	0.230	0.891
IL-17	10.4	1.7 (1.7–1.8)	34.7	1.7 (1.7–19.7)	0.007 *	0.305	0.035 *	0.133
TNF-α	83.3	24.6 (8.7–49.4)	81.6	35.8 (13.9–109.4)	1.000	0.606	0.070	0.805
Th1-related cytokines								
IL-12 (p70)	0	1.2 (1.2–1.2)	0	1.2 (1.2–1.2)	-	-	0.886	0.498
IFN-γ	56.3	2.7 (0.5–31.3)	77.6	16.9 (2.4–41.7)	0.032 *	0.868	0.067	0.995
OPN	97.9	318.1 (204.4–439.8)	93.8	137.5 (81.9–263.5)	0.617	0.503	0.00002 *	0.0016 *
Th2-related cytokines								
IL-4	50.0	0.4 (0.4–1.7)	55.1	0.8 (0.4–4.7)	0.686	0.357	0.316	0.454
IL-5	2.1	3.7 (3.7–3.9)	18.4	3.9 (3.7–3.9)	0.016 *	0.172	0.103	0.886
IL-9	25.0	2.5 (2.4–6.3)	42.9	2.5 (2.4–18.4)	0.087	0.251	0.214	0.166
IL-13	0	0.3 (0.3–0.3)	0	0.3 (0.3–0.3)	-	-	0.970	0.865
GM-CSF	8.3	0.4 (0.4–0.9)	14.3	0.4 (0.4–0.9)	0.524	0.576	0.466	0.856
Regulatory cytokines								
IL-10	2.1	1.7 (1.7–1.7)	4.1	1.7 (1.7–1.7)	1.000	0.485	0.718	0.724
TGF-β1	100	1781.7 (1331.7–2469.1)	100	1956.3 (1302.6–2497.6)	-	-	0.748	0.937
Chemokines								
IL-8	100	139.4 (39.2–532.1)	100	303.7 (118.3–1562.8)	-	-	0.009 *	0.838
Eotaxin	89.6	25.1 (1.9–76.2)	91.8	29.2 (2.7–55.9)	0.740	0.427	0.549	0.251
IP-10	100	7022.3 (2053.6–22,281.6)	100	10,884.9 (3134.3–20,592.8)	-	-	0.395	0.740
MCP-1	100	182.5 (77.7–715.0)	100	330.7 (165.4–798.1)	-	-	0.133	0.664
MIP-1α	95.8	2.5 (1.4–8.6)	100	9.8 (3.7–146.0)	0.242	0.683	0.0002 *	0.120
MIP-1β	97.9	32.6 (11.3–93.6)	100	52.6 (13.5–143.1)	0.495	1.000	0.113	0.788
RANTES	47.9	3.2 (3.2–29.6)	51.0	3.6 (3.2–45.2)	0.840	0.844	0.724	0.998
Growth factors								
IL-2	8.3	1.1 (1.0–1.1)	26.5	1.1 (1.0–1.3)	0.031 *	0.510	0.060	0.522
IL-7	50.0	10.2 (2.4–47.5)	55.1	12.9 (7.9–33.0)	0.686	0.574	0.775	0.561
IL-15	4.2	8.6 (8.4–8.8)	20.4	8.8 (8.4–9.2)	0.028 *	0.258	0.067	0.743
FGF-basic	8.3	3.7 (3.1–3.9)	32.7	3.7 (3.1–28.8)	0.005 *	0.492	0.024 *	0.086
G-CSF	91.7	87.3 (41.1–337.0)	91.8	314.8 (105.9–1909.4)	1.000	0.521	0.0003 *	0.167
PDGF-BB	10.4	10.5 (4.2–17.2)	24.5	15.9 (4.2–17.2)	0.108	0.779	0.246	0.765
VEGF	97.9	12,092.6 (6932.8–18,474.8)	100	16,621.5 (10,038.3–25,498.4)	0.495	1.000	0.078	0.661
Other								
sCD14	100	17.9 (12.4–22.8)	97.9	16.1 (12.8–20.6)	1.000	-	0.436	0.372

Abbreviations: IL-1ra, interleukin-1 receptor antagonist; IL-1β, interleukin-1β; IL-6, interleukin-6; IL-17, interleukin-17; TNF-α, tumor necrosis factor-α; IL-12 (p70), interleukin-12 (p70); IFN-γ, interferon-γ; OPN, osteopontin; IL-4, interleukin-4; IL-5, interleukin-5; IL-9, interleukin-9; IL-13, interleukin-13; GM-CSF, granulocyte macrophage colony-stimulating factor; IL-10, interleukin-10; TGF-β, transforming growth factor-β; IL-8, interleukin-8; IP-10, interferon-γ induced protein-10; MCP-1, monocyte chemotactic protein-1; MIP-1α, macrophage inflammatory protein-1α; MIP-1β, macrophage inflammatory protein-1β; RANTES, regulated on activation normal T cell expressed and secreted; IL-2, interleukin-2; IL-7, interleukin-7; IL-15, interleukin-15; FGF-basic, fibroblast growth factor-basic; G-CSF, granulocyte colony-stimulating factor; PDGF-BB, platelet-derived growth factor-BB; VEGF, vascular endothelial growth factor; sCD14, soluble CD14; IQR, interquartile range; C, colostrum; MM, mature milk. Median values are in pg/mL, except for those of OPN and sCD14, which are in μg/mL. The differences in positive rates between 1989 and 2013 were examined using Fisher’s exact test; after adjustment for “maternal age (year)” and “infant birth weight (g)” as predisposing variables, these differences were examined using a logistic regression (with the Firth correction method). The differences in cytokine levels between 1989 and 2013 were examined using the Mann–Whitney U test; after adjustment for “maternal age (year)” and “infant birth weight (g)” as the predisposing variables, these differences were examined using a multiple regression with rank order as the dependent variable. * *p* < 0.05.

**Table 3:** Cytokine profile in the MM samples.

	1989 (*n* = 48)		2013 (*n* = 49)		*p* Value			
	Positive Results (%)	Median (IQR)	Positive Results (%)	Median (IQR)	Fisher’s Exact Test	Logistic Regression	Mann–Whitney U Test	Multiple Regression
Anti-inflammatory cytokines								
IL-1ra	79.2	78.1 (25.4–206.9)	89.8	123.7 (44.1–242.8)	0.171	0.850	0.140	0.301
Proinflammatory cytokines								
IL-1β	31.3	0.1 (0.1–0.3)	36.7	0.1 (0.1–0.4)	0.669	0.256	0.344	0.270
IL-6	33.3	0.5 (0.5–4.9)	40.8	0.5 (0.4–4.3)	0.530	0.682	0.752	0.981
IL-17	2.1	1.7 (1.7–1.7)	4.1	1.7 (1.7–1.7)	1.000	0.226	0.863	0.668
TNF-α	52.1	3.5 (2.4–20.0)	38.8	3.1 (2.4–17.3)	0.224	0.214	0.429	0.247
Th1-related cytokines								
IL-12 (p70)	0	1.2 (1.2–1.2)	0	1.2 (1.2–1.2)	-	-	0.941	0.515
IFN-γ	16.7	0.5 (0.4–1.7)	30.6	0.5 (0.4–3.0)	0.152	0.227	0.118	0.071
OPN	100	300.8 (208.4–344.6)	98.0	280.9 (220.6–337.9)	1.000	1.000	0.697	0.391
Th2-related cytokines								
IL-4	8.3	0.1 (0.1–0.4)	10.2	0.4 (0.1–0.4)	1.000	0.508	0.545	0.437
IL-5	0	3.7 (3.7–3.9)	2.0	3.7 (3.7–3.9)	1.000	1.000	0.856	0.263
IL-9	6.3	2.5 (2.4–2.5)	10.2	2.5 (2.4–2.5)	0.715	0.538	0.652	0.553
IL-13	0	0.3 (0.3–0.3)	0	0.3 (0.3–0.3)	-	-	0.943	0.477
GM-CSF	12.5	0.4 (0.4–0.9)	2.0	0.4 (0.4–0.4)	0.059	0.709	0.204	0.794
Regulatory cytokines								
IL-10	0	1.7 (1.7–1.7)	0	1.7 (1.7–1.7)	-	-	0.916	0.907
TGF-β1	100	1056.2 (813.9–1720.5)	100	1330.8 (988.5–1809.9)	-	-	0.274	0.008 *
Chemokines								
IL-8	100	36.8 (14.2–82.8)	100	42.1 (20.7–91.4)	-	-	0.421	0.183
Eotaxin	58.3	0.7 (0.1–8.2)	42.9	0.1 (0.1–2.1)	0.157	0.089	0.079	0.111
IP-10	93.8	1717.0 (185.9–7154.8)	89.8	432.4 (154.9–2779.2)	0.715	0.235	0.023 *	0.124
MCP-1	93.8	44.3 (9.0–105.5)	93.9	58.7 (18.2–180.0)	1.000	0.941	0.256	0.628
MIP-1α	68.8	1.1 (0.1–3.2)	65.3	1.6 (0.1–5.7)	0.830	0.272	0.595	0.832
MIP-1β	77.1	7.1 (0.5–17.8)	67.3	5.4 (0.5–21.1)	0.366	0.184	0.699	0.735
RANTES	12.5	3.2 (2.9–3.2)	8.2	3.2 (2.9–3.2)	0.524	0.643	0.991	0.867
Growth factors								
IL-2	2.1	1.1 (1.0–1.1)	2.0	1.1 (1.0–1.1)	1.000	0.089	0.668	0.913
IL-7	25.0	7.9 (2.4–11.4)	14.3	7.9 (2.3–9.1)	0.210	0.017 *	0.285	0.279
IL-15	2.1	8.6 (8.4–8.8)	2.0	8.6 (8.4–8.8)	1.000	0.089	0.837	0.355
FGF-basic	2.1	3.7 (3.1–3.7)	4.1	3.7 (3.1–3.7)	1.000	0.226	0.805	0.679
G-CSF	47.9	7.2 (6.8–109.9)	53.1	50.9 (7.0–174.1)	0.686	0.870	0.485	0.333
PDGF-BB	0	5.0 (4.2–15.9)	6.1	5.0 (4.2–17.2)	0.242	0.060	0.347	0.340
VEGF	97.9	3003.3 (1613.9–4707.2)	100	2470.7 (2131.6–3507.7)	0.495	1.000	0.660	0.759
Other								
sCD14	97.9	9.8 (5.9–14.5)	100	8.0 (6.4–11.5)	0.495	1.000	0.614	0.269

Abbreviations: IL-1ra, interleukin-1 receptor antagonist; IL-1β, interleukin-1β; IL-6, interleukin-6; IL-17, interleukin-17; TNF-α, tumor necrosis factor-α; IL-12 (p70), interleukin-12 (p70); IFN-γ, interferon-γ; OPN, osteopontin; IL-4, interleukin-4; IL-5, interleukin-5; IL-9, interleukin-9; IL-13, interleukin-13; GM-CSF, granulocyte macrophage colony-stimulating factor; IL-10, interleukin-10; TGF-β, transforming growth factor-β; IL-8, interleukin-8; IP-10, interferon-γ induced protein-10; MCP-1, monocyte chemotactic protein-1; MIP-1α, macrophage inflammatory protein-1α; MIP-1β, macrophage inflammatory protein-1β; RANTES, regulated on activation normal T cell expressed and secreted; IL-2, interleukin-2; IL-7, interleukin-7; IL-15, interleukin-15; FGF-basic, fibroblast growth factor-basic; G-CSF, granulocyte colony-stimulating factor; PDGF-BB, platelet-derived growth factor-BB; VEGF, vascular endothelial growth factor; sCD14, soluble CD14; IQR, interquartile range; C, colostrum; MM, mature milk. Median values are in pg/mL, except for those of OPN and sCD14, which are in μg/mL. The differences in positive rates between 1989 and 2013 were examined using Fisher’s exact test; after adjustment for “maternal age (year)” and “infant birth weight (g)” as predisposing variables, these differences were examined using a logistic regression (with the Firth correction method). The differences in cytokine levels between 1989 and 2013 were examined using the Mann–Whitney U test; after adjustment for “maternal age (year)” and “infant birth weight (g)” as the predisposing variables, these differences were examined using a multiple regression with rank order as the dependent variable. * *p* < 0.05.

**Table 4:** Lactational change ratios of cytokines (MM/C).

	Cytokine Ratios (MM/C)		
	1989 (*n* = 48)	2013 (*n* = 49)	*p* Value
Proinflammatory cytokines			
IL-1β	0.57	0.14	0.021 *
TNF-α	0.35	0.15	0.039 *
Th1-related cytokines			
IFN-γ	0.53	0.07	0.269
OPN	0.87	1.92	0.0009 *
Th2-related cytokines			
IL-4	0.90	0.21	0.363
Chemokines			
IL-8	0.26	0.11	0.111
Eotaxin	0.08	0.02	0.180
IP-10	0.31	0.04	0.007 *
MIP-1α	0.22	0.04	0.012 *
Growth factors			
G-CSF	0.16	0.07	0.120

Abbreviations: IL-1β, interleukin-1β; TNF-α, tumor necrosis factor-α; IFN-γ, interferon-γ; OPN, osteopontin; IL-4, interleukin-4; IL-8, interleukin-8; IP-10, interferon-γ induced protein-10; MIP-1α, macrophage inflammatory protein-1α; G-CSF, granulocyte colony-stimulating factor; MM, mature milk; C, colostrum. Cytokines were selected for this table if their ratios were two-fold and above or less than half-fold. The median values represent cytokine ratios (MM/C). The ratios between the two groups were analyzed using the Mann–Whitney U test. * *p* < 0.05.

## Data Availability

The datasets used in this study are not publicly available, as they contain personal information. Requests for access to these datasets should be directed to Tomoki Takahashi (tomoki-takahashi@beanstalksnow.co.jp).

## Supplementary Materials

The following supporting information can be downloaded at https://www.mdpi.com/article/10.3390/nu15071735/s1, Table S1: Adjusted odds ratios (1989/2013) for positive results of cytokines in colostrum and mature milk., Table S2: Breast milk OPN levels and background characteristics of mothers and infants who experienced vaginal delivery or c-section in 2013 study.
